# Supplementary material for: Rapid and Recent Evolution of LTR Retrotransposons Drives Rice Genome Evolution During the Speciation of AA-Genome Oryza Species
Source: G3 (Bethesda). 2017 Apr 14;7(6):1875–85. doi: 10.1534/g3.116.037572 (PMC5473765; doi:10.1534/g3.116.037572)
Supplement: Supplementary file 1 [file 1875FileS1.docx]

**Rapid and recent evolution of LTR retrotransposons drives rice genome evolution during the speciation of AA- genome *Oryza* species**

**Qun-Jie Zhang ^1, 2, 3^, and Li-Zhi Gao^1, *^**

^1^ Plant Germplasm and Genomics Center, Kunming Institute of Botany, the Chinese Academy of Sciences, Kunming 650204, China

^2^ University of the Chinese Academy of Sciences, Beijing 100039, China

^3^Agrobiological Gene Research Center, Guangdong Academy of Agricultural

Sciences, Guangzhou 510640, China

* **Corresponding Author:**

Li-zhi Gao

Tel./Fax: (+0086871) -65223277

E-mail: Lgao@mail.kib.ac.cn

**Running title:**

Recent and rapid evolution of rice LTR retrotransposons

**Keywords:**

LTR retrotransposons; *Oryza*; AA- genomes; rice speciation; comparative genomics

**Supplementary Information**


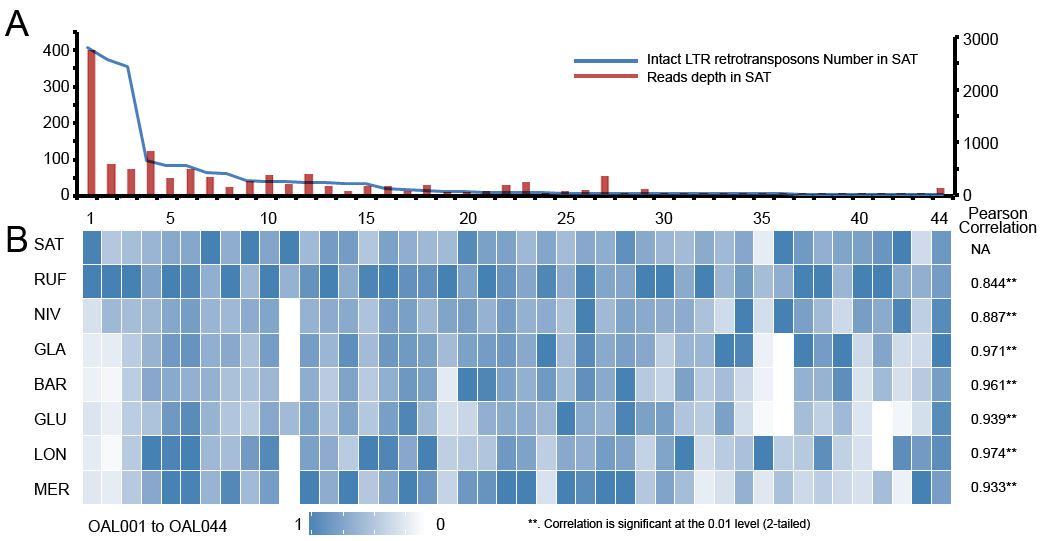


**Figure S1. Comparison of the top forty-four multi-copy LTR retrotransposon families across the eight AA- genome *Oryza* species.** (**A**) LTR reads depth and numbers of intact elements in SAT. The scale on left indicates the number of intact LTR retrotransposons in SAT (blue line), and the scale on right is reads depth in SAT (red bars). (**B**) Heatmap of proportions of LTR reads depth within the families across the eight rice genomes. Pearson correlations were calculated between reads depth for SAT and that for each of the seven non-SAT species, respectively.


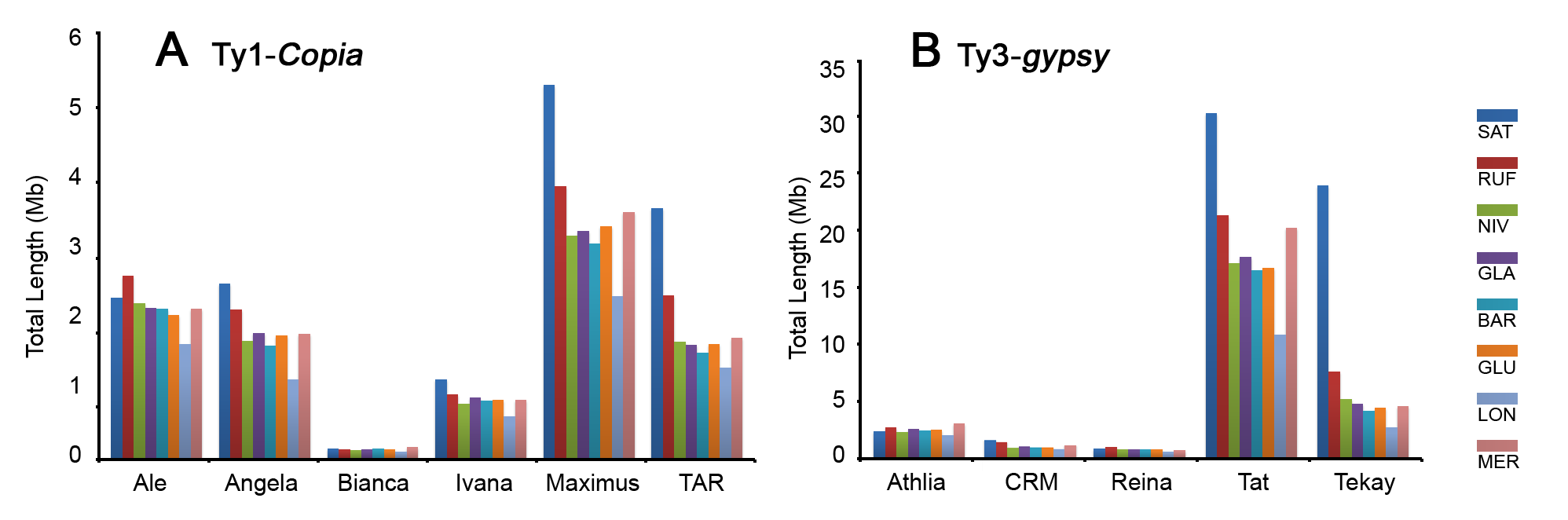


**Figure S2. Statistic of the Ty1-*copia* and Ty3-*gypsy* families across the eight AA- genome *Oryza* species.** Total lengths of Ty1-*copia* (**A**) and Ty3-*gypsy* (**B**) elements across the eight AA-genome *Oryza* species by using RepeatMasker.

**
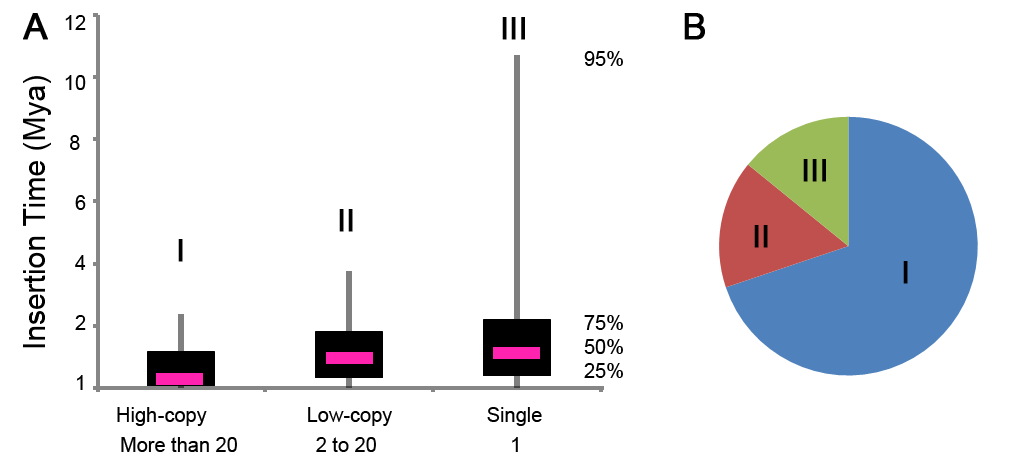
**

**Figure S3. Comparisons of insertion times and sequence length proportions of LTR retrotransposon families classified by copy number in SAT.** (**A**) Insertion times of the three different types of LTR retrotransposon families (I: more than 20 copies; II: 2-20 copies; III: one copy). Red sports indicate mean values, black bars show the ranges varying between 25% and 75%, and grey bars represent ranges ranging from 5% to 95%. (**B**) Proportions of total sequence lengths of these three types of LTR retrotransposon families.

**
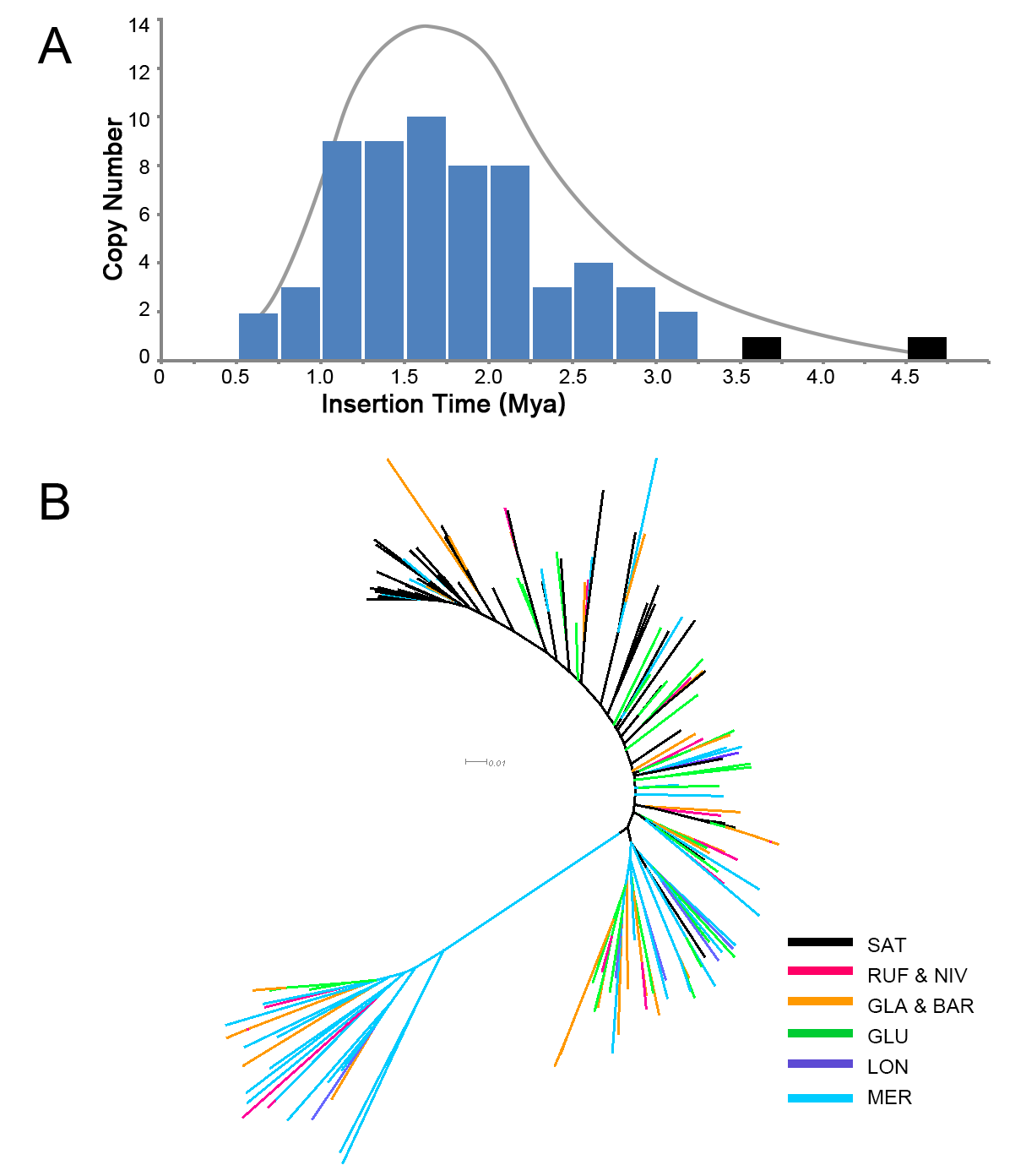
**

**Figure S4. Evolutionary dynamics of the family OAL008 across the eight AA- genome *Oryza* species.** (**A**) Distribution of insertion times. Blue bars show the inserted copies in SAT, and black bars indicate the two older insertion events in other AA- genome *Oryza* species. The gray line represents insertion curve amended by half-life as 3 - 4 Myr. (**B**) Phylogenetic tree constructed by using RTs of the OAL008 family across the eight AA- genome *Oryza* species.

**Table S1. Statistics of intact elements of the top forty-four LTR retrotransposon families in the eight AA- genome *Oryza* species.**

| **Family** | **S*** | **Total** | **AA- genome *Oryza* Species** | | | | | | | | **Age (Myr)**** |
| --- | --- | --- | --- | --- | --- | --- | --- | --- | --- | --- | --- |
| **ID** |  | **No.** | **SAT** | **RUF** | **NIV** | **GLA** | **BAR** | **GLU** | **LON** | **MER** | 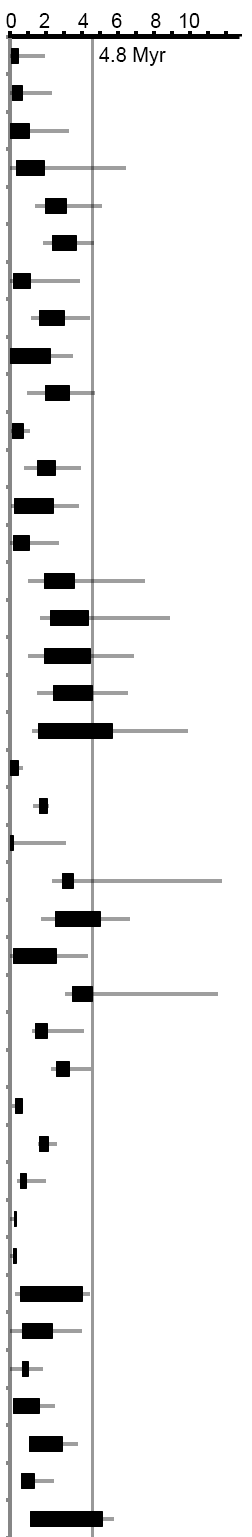 |
| OAL001 | **g** | 413 | 408 | 1 | 1 | 0 | 0 | 0 | 2 | 1 |  |
| OAL002 | **g** | 383 | 374 | 3 | 0 | 4 | 1 | 0 | 1 | 0 |  |
| OAL003 | **g** | 390 | 255 | 16 | 3 | 3 | 2 | 0 | 7 | 4 |  |
| OAL004 | **n** | 121 | 97 | 7 | 4 | 2 | 1 | 5 | 4 | 1 |  |
| OAL005 | **g** | 108 | 85 | 9 | 3 | 1 | 2 | 1 | 5 | 2 |  |
| OAL006 | **g** | 113 | 84 | 9 | 2 | 2 | 1 | 2 | 13 | 0 |  |
| OAL007 | **c** | 70 | 65 | 1 | 0 | 1 | 1 | 0 | 2 | 0 |  |
| OAL008 | **g** | 88 | 63 | 6 | 3 | 5 | 1 | 1 | 6 | 3 |  |
| OAL009 | **c** | 47 | 44 | 1 | 0 | 1 | 0 | 0 | 1 | 0 |  |
| OAL010 | **c** | 42 | 41 | 0 | 0 | 0 | 0 | 0 | 1 | 0 |  |
| OAL011 | **g** | 40 | 40 | 0 | 0 | 0 | 0 | 0 | 0 | 0 |  |
| OAL012 | **g** | 49 | 38 | 6 | 1 | 0 | 0 | 0 | 4 | 0 |  |
| OAL013 | **c** | 39 | 37 | 1 | 1 | 0 | 0 | 0 | 0 | 0 |  |
| OAL014 | **c** | 36 | 35 | 1 | 0 | 0 | 0 | 0 | 0 | 0 |  |
| OAL015 | **g** | 36 | 34 | 0 | 0 | 0 | 0 | 0 | 2 | 0 |  |
| OAL016 | **n** | 62 | 20 | 7 | 8 | 7 | 6 | 7 | 2 | 5 |  |
| OAL017 | **c** | 41 | 17 | 8 | 3 | 3 | 2 | 2 | 4 | 2 |  |
| OAL018 | **g** | 23 | 14 | 3 | 1 | 1 | 0 | 0 | 3 | 1 |  |
| OAL019 | **n** | 47 | 13 | 7 | 6 | 7 | 4 | 3 | 1 | 6 |  |
| OAL020 | **c** | 25 | 13 | 2 | 1 | 3 | 5 | 1 | 0 | 0 |  |
| OAL021 | **c** | 11 | 11 | 0 | 0 | 0 | 0 | 0 | 0 | 0 |  |
| OAL022 | **g** | 15 | 9 | 2 | 1 | 0 | 0 | 1 | 2 | 0 |  |
| OAL023 | **g** | 14 | 9 | 3 | 0 | 0 | 0 | 0 | 2 | 0 |  |
| OAL024 | **c** | 9 | 9 | 0 | 0 | 0 | 0 | 0 | 0 | 0 |  |
| OAL025 | **n** | 10 | 8 | 2 | 0 | 0 | 0 | 0 | 0 | 0 |  |
| OAL026 | **c** | 11 | 7 | 1 | 0 | 0 | 0 | 0 | 3 | 0 |  |
| OAL027 | **g** | 11 | 7 | 2 | 0 | 0 | 1 | 0 | 1 | 0 |  |
| OAL028 | **n** | 10 | 7 | 1 | 0 | 0 | 1 | 0 | 1 | 0 |  |
| OAL029 | **c** | 7 | 7 | 0 | 0 | 0 | 0 | 0 | 0 | 0 |  |
| OAL030 | **g** | 7 | 7 | 0 | 0 | 0 | 0 | 0 | 0 | 0 |  |
| OAL031 | **n** | 26 | 6 | 4 | 1 | 3 | 2 | 3 | 5 | 2 |  |
| OAL032 | **n** | 26 | 6 | 4 | 3 | 3 | 4 | 4 | 1 | 1 |  |
| OAL033 | **c** | 17 | 6 | 1 | 3 | 1 | 2 | 2 | 0 | 2 |  |
| OAL034 | **c** | 10 | 6 | 2 | 0 | 1 | 1 | 0 | 0 | 0 |  |
| OAL035 | **c** | 8 | 6 | 2 | 0 | 0 | 0 | 0 | 0 | 0 |  |
| OAL036 | **c** | 8 | 6 | 1 | 0 | 0 | 0 | 0 | 0 | 1 |  |
| OAL037 | **c** | 29 | 5 | 5 | 3 | 4 | 4 | 6 | 0 | 2 |  |
| OAL038 | **n** | 25 | 5 | 4 | 5 | 3 | 1 | 3 | 0 | 4 |  |
| OAL039 | **c** | 16 | 5 | 1 | 3 | 3 | 3 | 1 | 0 | 0 |  |
| OAL040 | **g** | 14 | 5 | 3 | 3 | 1 | 1 | 0 | 0 | 1 |  |
| **Family** | **S*** | **Total** | **AA- genome *Oryza* Species** | | | | | | | | **Age (Myr)*** |
| **ID** |  | **No.** | **SAT** | **RUF** | **NIV** | **GLA** | **BAR** | **GLU** | **LON** | **MER** | 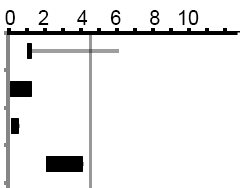 |
| OAL041 | **g** | 9 | 5 | 1 | 0 | 1 | 1 | 0 | 0 | 1 |  |
| OAL042 | **c** | 6 | 5 | 0 | 0 | 1 | 0 | 0 | 0 | 0 |  |
| OAL043 | **g** | 6 | 5 | 0 | 0 | 0 | 1 | 0 | 0 | 0 |  |
| OAL044 | **c** | 5 | 5 | 0 | 0 | 0 | 0 | 0 | 0 | 0 |  |

* represents super-family, 'g' symbolizes *gypsy*-like families, 'c' indicates *copia*-like families, and 'n' denotes unclassified families.

** Black bars show the ranges between 25% and 75%, gray bars represent the ranges from 5% to 95%, and grey vertical lines designate the divergence times between SAT and MER; Myr: million years ago.

**Supplementary table S2 LTR retrotransposon families that correspond to the previously reported names.**

| **Family ID** | **Previously reported name** |
| --- | --- |
| OAL001 | *RIRE3, RIRE8, osr33, osr34,* vobad, wube, veiko, seaba |
| OAL002 | *Hopi, osr27*, ovar, ifisi, ofon, noedu, oren |
| OAL003 | *Dasheng, RIRE2, osr25, osr26* |
| OAL004 | osr37, noaCRR1, CRR2 |
| OAL005 | RIRE10, osr40, sipy |
| OAL006 | pawepe, noaCRR2 |
| OAL007 | *Houba*, *osr13* |
| OAL008 | *osr41*, vobad |
| OAL009 | *osr17* |
| OAL010 | *osr8*, *osr7* |
| OAL011 | *Dagul,* park, eguag |
| OAL012 | *osr30* |
| OAL013 | *osr1, osr5, RIRE5* |
| OAL014 | *osr4*, ulamen |
| OAL015 | *osr29*, ovikoh |
| OAL016 | *osr15* |
| OAL018 | pese |
| OAL019 | *osr3* |
| OAL020 | echidne |
| OAL021 | ohutu, gileub |
| OAL023 | awok |
| OAL026 | *osr14* |
| OAL028 | wuvu |
| OAL029 | *osr10* |
| OAL030 | *RIRE7* |
| OAL031 | agab |
| OAL032 | *osr16* |
| OAL033 | *osr6* |
| OAL034 | *osr12* |
| OAL035 | ekop |
| OAL039 | *osr24* |
| OAL041 | *osr36, osr39*, ofava |
| OAL045 | jyfu |
| OAL052 | *osr43* |
| OAL053 | *osr22* |
